# A Pol V–Mediated Silencing, Independent of RNA–Directed DNA Methylation, Applies to 5S rDNA

**DOI:** 10.1371/journal.pgen.1000690

**Published:** 2009-10-16

**Authors:** Julien Douet, Sylvie Tutois, Sylvette Tourmente

**Affiliations:** CNRS, UMR 6247 GReD, Clermont Université, INSERM U931, Aubière, France; The Salk Institute for Biological Studies, United States of America

## Abstract

The plant-specific RNA polymerases Pol IV and Pol V are essential to RNA–directed DNA methylation (RdDM), which also requires activities from RDR2 (RNA–Dependent RNA Polymerase 2), DCL3 (Dicer-Like 3), AGO4 (Argonaute), and DRM2 (Domains Rearranged Methyltransferase 2). RdDM is dedicated to the methylation of target sequences which include transposable elements, regulatory regions of several protein-coding genes, and 5S rRNA–encoding DNA (rDNA) arrays. In this paper, we have studied the expression of the 5S-210 transcript, a marker of silencing release at 5S RNA genes, to show a differential impact of RNA polymerases IV and V on 5S rDNA arrays during early development of the plant. Using a combination of molecular and cytological assays, we show that Pol IV, RDR2, DRM2, and Pol V, actors of the RdDM, are required to maintain a transcriptional silencing of 5S RNA genes at chromosomes 4 and 5. Moreover, we have shown a derepression associated to chromatin decondensation specific to the 5S array from chromosome 4 and restricted to the Pol V–loss of function. In conclusion, our results highlight a new role for Pol V on 5S rDNA, which is RdDM–independent and comes specifically at chromosome 4, in addition to the RdDM pathway.

## Author Summary

In plant genomes, the RNA–directed DNA methylation (RdDM) process induces *de novo* methylation of cytosines at repeated sequences. The RNA polymerases Pol IV and Pol V are two key components of the RdDM pathway. Pol IV acts with RDR2 (RNA–dependent RNA polymerase 2) and DCL3 (Dicer-Like protein 3) to generate short interfering RNAs (siRNAs). Pol V, in a partnership including AGO4 (Argonaute4) and DRM2 (Domains Rearranged Methyltransferase 2), drives DNA methylation at the targeted sequence. Changes in 5S (ribosomal DNA) rDNA methylation, 5S rDNA chromatin compaction, and 5S siRNA accumulation in Pol IV/V mutants have been reported. However, 5S rDNA arrays were considered together. In the present study, we observed an overexpression of the atypic 5S-210 transcript, restricted to the 5S rDNA array from chomosome 4. This derepression is specific to the Pol V–loss of function (and not to Pol IV) and comes in addition to the RdDM pathway. The Pol V–loss of function induces also the chromatin decondensation of the derepressed 5S locus at chomosome 4. Our results highlight a new role for Pol V which, suprisingly, appears to be Pol IV– and RdDM–independent.

## Introduction

The plant-specific RNA polymerases firstly named Pol IVa and Pol IVb and now referred as Pol IV and Pol V [1], contribute to siRNA production and are essential to RNA-directed DNA methylation (RdDM) [2]–[6]. The revised nomenclature denotes the largest subunits of Pol IV and Pol V as NRPD1 and NRPE1. Both Pol IV and Pol V share the second largest subunit NRPD2. In current models of RdDM silencing pathway [7]–[10], Pol IV is speculated to produce single-stranded RNA transcripts from heterochromatic repeated regions. These transcripts, converted onto double-stranded RNAs by RDR2 (RNA-DEPENDENT RNA POLYMERASE 2), are processed into siRNA duplexes by DCL3 (DICER-LIKE 3) [11]–[14]. The resulting siRNAs are methylated at 2′ hydroxyl groups of 3′-terminal nucleotides by HEN1 (HUA ENHANCER 1) prior to loading into AGO4/RISC complex [15]. Recently, Pol V and DRD1 (DEFECTIVE IN RNA-DIRECTED DNA METHYLATION 1) were found to be required to mediate production of non-coding transcripts which are necessary for steps downstream of siRNA biogenesis [1]. Recent evidence suggests that siRNAs/AGO4 complexes bind to Pol V transcripts, ultimately guiding the *de novo* DNA methyltransferase DRM2 [16] and histone modifying complexes to the target loci [17],[18].

Targets of the Pol IV/Pol V-dependent RdDM include transposable elements, regulatory regions of several protein-coding genes and 5S rRNA-encoding DNA (rDNA) arrays [2]–[5], [19]–[23].

We [24] and others [4],[5],[11] have reported changes in 5S rDNA methylation, 5S rDNA chromatin compaction and 5S siRNA accumulation in Pol IV/V mutants. However, in these reports, 5S rDNA arrays, which have separate functions and locations (For a review, [25]) were considered together.

*Arabidopsis thaliana* contains approximately 1000 copies of 5S RNA genes per haploid genome. 5S rDNA is arranged in tandem arrays [26] located within the pericentromeric heterochromatin of chromosomes 3, 4 and 5 in the Columbia accession [27],[28]. Only 5S-repeat clusters located on chromosomes 4 and 5 are transcribed by Pol III to produce the 120 nucleotide (nt) transcripts which are integrated into ribosomes [29]. Nevertheless, it is not yet clear what proportion of these 5S genes is active at any one time.

Indeed, both active 5S-repeat clusters contain transcribed and repressed 5S RNA genes in WT plants. Previous study revealed that in adult wild-type plants, only «major» 5S RNA genes were expressed whereas «minor» genes, which diverge from «major» ones at only one or several positions, are repressed [30]. In addition to «major» and «minor» 5S RNA species, we have previously identified an atypical 5S RNA (5S-210) composed of genic and intergenic regions which is a marker of silencing release at 5S RNA genes [31]. The presence of a chromosome-specific T stretch identifies the chromosome origin of 5S-210 transcripts [29].

To determine the individual contribution of Pol IV and Pol V in the transcriptional silencing and heterochromatic state of each 5S array, we assayed 5S-210 transcript expression, chromatin compaction and DNA methylation during early development. We have shown that Pol IV, Pol V and several actors of the RdDM, contribute to maintain a transcriptional silencing of 5S RNA genes at chromosomes 4 and 5. Moreover, we showed an additional Pol V activity, Pol IV- and RdDM-independent which drives silencing of 5S rDNA specifically at chromosome 4. The large silencing release observed at chromosome 4 in NRPE1 and NRPE5a (Pol V) mutants, is accompanied by a decompaction of the corresponding 5S rDNA locus, as well as decompaction of NOR loci.

## Results

### Different impact of Pol IV and Pol V on 5S RNA genes silencing

**Figure 1:**
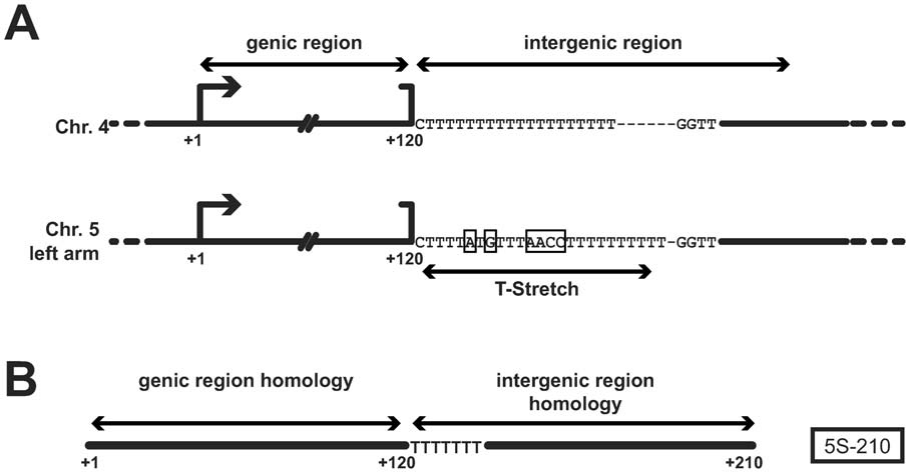
Schematic representation of a 5S rDNA unit and 5S-210 transcript. (A) A 5S rDNA unit contains the 120 bp-genic region followed by the intergenic region containing the 5S array-specific T-stretch. Boxes represent differences between the T-stretch from chromosome 4 and chromosome 5 left arm. (B) The 5S-210 transcript is homologous to the 120 nt-genic region and 90 nt from the adjacent intergenic region.

We previously identified a 5S transcript, 210 bases-long (5S-210) which is a marker of silencing release at 5S RNA genes. This 5S-210 transcript homologous to the 120 nt genic region and 90 nt from the adjacent intergenic region [31] contains the sequence of the chromosome-specific T-stretch which identifies its 5S array-origin (Figure 1).

In order to define the impact of Pol IV (formerly Pol IVa) and Pol V (formerly Pol IVb) on 5S RNA genes silencing, we analysed 5S-210 accumulation by RT-PCR experiments in WT, *nrpd1* (mutant of the largest subunit of Pol IV), *nrpd2* (mutant of the common subunit of Pol IV and Pol V), *nrpe1* (mutant of the largest subunit of Pol V) and *nrpe5a* (mutant of a new Pol V-specific subunit; [32]) plants. RDR2 (RNA-DEPENDENT RNA POLYMERASE 2), DCL3 (DICER-LIKE 3), AGO4 (ARGONAUTE 4), DRM2 (DOMAINS REARRANGED METHYLTRANSFERASE 2), HEN1 (HUA ENHANCER 1) and HDA6 (HISTONE DEACETYLASE 6) involved with Pol IV and POL V in the production of siRNAs and the associated DNA methylation and histone modifications, were also tested.

**Figure 2:**
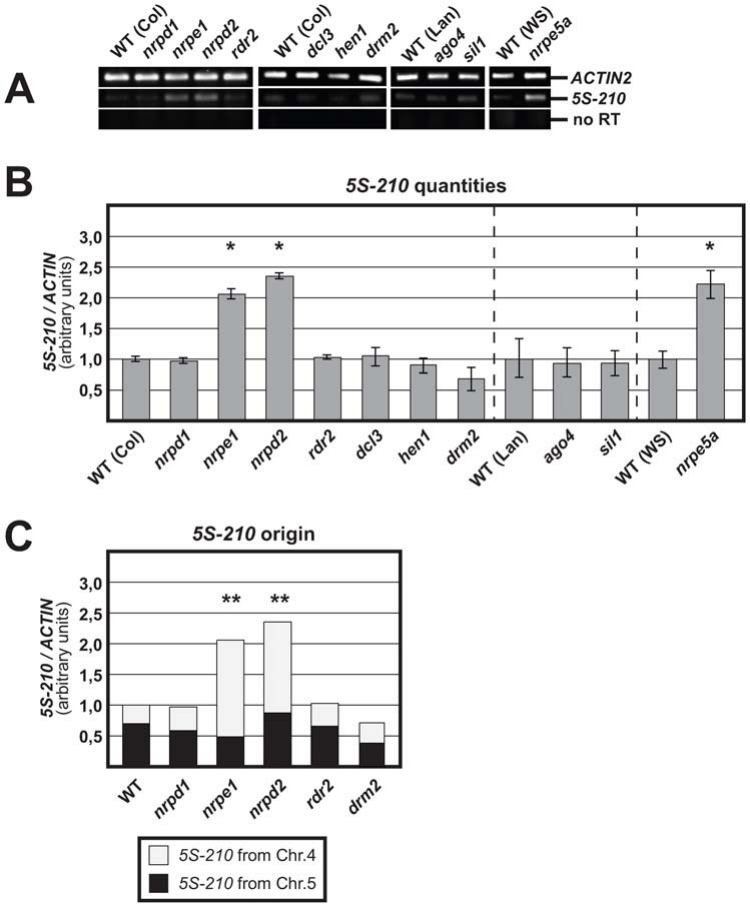
RT–PCR detection of 5S-210 transcripts in WT, *nrpd1*, *nrpe1*, *nrpd2*, *rdr2*, *dcl3*, *hen1*, *drm2*, *ago4*, *sil1*, and *nrpe5a* plants. (A) Expression of *ACTIN 2* was used to normalize the *5S-210* amounts. Negative controls were performed without reverse transcriptase (no RT). (B) A graphic representation of 5 to 7 independent experiments is given. The standard deviation of the mean is indicated on each bar. Asterisks indicate values significantly different from the WT value which is arbitrarily fixed as 1 (Mann-Whitney test, * P<0.05). (C) Proportion of 5S-210 transcripts from chromosomes 4 and 5. RT-PCR products from (B) were sequenced and the T-stretch signature analysed to determine the 5S-array' origin of 5S-210 transcripts. A total of 70, 53, 68, 51, 39, and 52 5S-210 cDNA clones were sequenced for WT, *nrpd1*, *nrpe1*, *nrpd2*, *rdr2* and *drm2* respectively. Statistics were done to appreciate the participation of 5S array from chromosome 4. Asterisks denote significant differences to the WT value (Fisher's exact test, ** P<0.001).

5S-210 transcripts overaccumulated by a factor between 2 and 2,5 in *nrpe1*, *nrpd2* and *nrpe5a* compared to WT plants. On the contrary, *nrpd1*, *rdr2*, *dcl3*, *hen1*, *ago4*, *drm2 and sil1* (mutant allele of HDA6) plants accumulate similar 5S-210 transcripts quantities than WT (Figure 2A and 2B).

The release of silencing in *nrpe1* and *nrpe5a* indicates that Pol V is involved in the quantitative regulation of 5S-210 expression. The results obtained with *nrpd2* confirm these observations. The absence of silencing release in *nrpd1*, *rdr2*, *dcl3*, *ago4*, *drm2*, *hen1* and *sil1* shows that they have no influence, at the quantitative level, on 5S-210 RNA transcription.

### Pol V mediates repression of 5S RNA genes from chromosome 4

5S rDNA arrays are located within the pericentromeric heterochromatin of chromosomes 3, 4 and 5. We assessed whether this Pol V-mediated silencing operates on all 5S arrays or operates selectively. Using the T stretch signature [29], we analysed the origin of the 5S-210 transcripts in Pol IV/Pol V mutants and in two mutants of proteins involved in respectively upstream and downstream RdDM steps *i.e.* RDR2 and DRM2. Sequencing of RT-PCR products revealed that 5S-210 transcripts only originate from the transcriptionally active 5S-repeat clusters located on chromosomes 4 and 5. In WT conditions, the 5S array from chromosome 5 contributes for 70%, the 5S array from chromosome 4 contributing for the remaining 30% of the 5S transcripts (Figure 2C). The proportions and quantities are not significantly different in *nrpd1*, *rdr2* and *drm2* compared to WT. In *nrpe1* and *nrpd2*, the overaccumulation of 5S-210 transcripts results only from the silencing release of the 5S array from chromosome 4, since the quantity of 5S-210 transcripts provided by chromosome 5 is unchanged (Figure 2C).

These results refine our previous conclusions indicating that the silencing release observed in *nrpe1* concerns the 5S array from chromosome 4; the results obtained with *nrpd2* confirm these observations. Therefore, the Pol V-mediated repression operates on the 5S array from chromosome 4.

### Pol V drives compaction of 5S rDNA from chromosome 4

**Figure 3:**
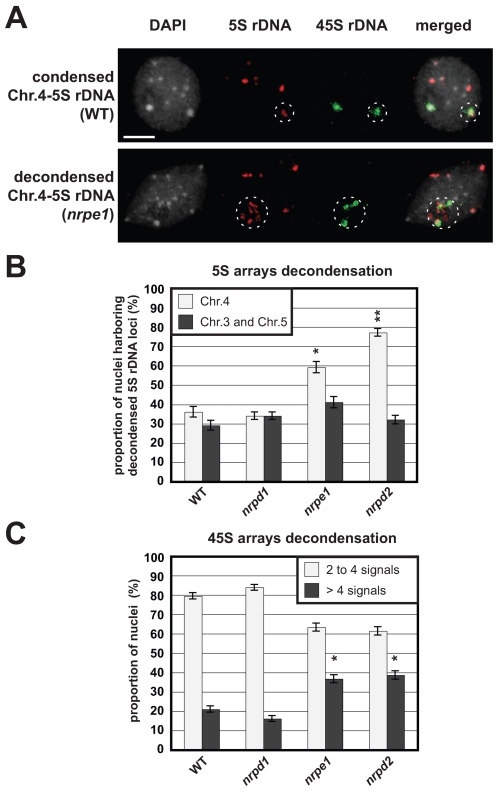
Compaction of rDNA arrays. (A) Representative images of condensed and decondensed 5S rDNA locus from chromosome 4. Counterstaining with DAPI (left); FISH with a 5S rDNA probe (red); FISH with a 25S rDNA probe which reveals the 45S locus (green); and the merged image on nuclei from WT and *nrpe1* nuclei. Associated 5S and 45S rDNA loci from chromosome 4 are circled. In both cases (WT and *nrpe1*), 5S signals from the two chromosomes 4 are present in the white circle. Non circled 5S-red signals are from chromosomes 3 and 5. Bar = 5 µm. B) Proportion of nuclei, derived from 45 to 62 nuclei analysed, harboring decondensed 5S signals. 5S signals from chromosome 4 were analysed separately whereas 5S signals from chromosomes 3 and 5 were considered together. Asterisks denote significant differences to the corresponding (chromosome 4 or 3 and 5) WT value. (Z-test, * P<0,05 , ** P<0,01). Interval confidence with a confidence level of 99% is shown on each bar. (C) The percentage of nuclei harboring 2 to 4 NOR loci (grey) and more than 4 NOR loci (black) was estimated from 45 to 62 nuclei analysed in WT, *nrpd1*, *nrpe1* and *nrpd2* plants. Asterisks denote significant differences to the WT value. (Z-test,* P<0,05). Interval confidence with a confidence level of 99% is shown on each bar.

In order to determine whether chromatin decompaction is associated to the release of silencing observed at chromosome 4, we performed FISH experiments with both 5S rDNA and 45S rDNA probes on WT, *nrpd1*, *nrpe1* and *nrpd2* plants. The chromosome 4 is the only chromosome to carry both rDNA species. 5S rDNA from chromosome 4 colocalizes with 45S rDNA signals in almost all nuclei [33]. 5S signals outside chromosome 4, *i.e.* coming from chromosomes 3 and 5 were considered together (Figure 3A and 3B).

FISH analysis revealed that 36% of the WT nuclei contain one or two decondensed 5S signals at chromosome 4. The proportions are similar in WT and *nrpd1* mutant whereas a significant larger proportion (59%) of the *nrpe1* and (77%) of *nrpd2* nuclei harbor decondensed 5S signals at chromosome 4. From these analyses, we conclude that 5S arrays from chromosome 4 are decondensed in *nrpe1*, and these results are confirmed with *nrpd2* observations, whereas *nrpd1* has no visible effect on this compaction. Therefore, NRPE1/Pol V has the ability to act on the chromatin of 5S rDNA from chromosome 4 in a NRPD1/Pol IV- independent manner.

We observed no significant variation in the proportion of nuclei with decompacted 5S signals at chromosomes 3 and 5 in WT, *nrpd1*, *nrpe1* and *nrpd2* plants (Figure 3B). These results show that *nrpd1* and *nrpe1* mutations have no visible effect on chromatin compaction of 5S arrays from chromosomes 3 and 5. From these analyses, we conclude there is a correlation between the large silencing release and the decompaction of 5S rDNA at chromosome 4 in *nrpe1* mutant.

### Pol V drives compaction of 45S rDNA

A question arises from these results: how and why is the 5S rDNA array from chromosome 4 concerned by this particular Pol V regulation? The main difference between 5S arrays from chromosomes 4 and 5 is the close proximity of NOR4 (Nucleolar Organizing Region) with the 5S array from chromosome 4. Therefore, we hypothesized that both rDNA arrays (5S and 45S) might be co-regulated and be the site of common decompaction events.

We analyzed the NOR condensation in WT, *nrpd1*, *nrpe1* and *nrpd2* mutants (Figure 3A and 3C). There are four NORs in diploid *A. thaliana*, but they tend to coalesce and in agreement with Pontes *et al.* [34] we detected two to four NORs in 79% of WT nuclei and more than four signals in 21% of them. Similar results were obtained in *nrpd1* nuclei. Contrarily, more than four NORs FISH signals were observed in 37% and 39% of *nrpe1* and *nrpd2* nuclei reflecting a significant increase of NOR decompaction compared to WT and *nrpd1* nuclei.

These results show that no detectable chromatin decompaction is observed for NOR loci and 5S array from chromosome 4 in *nrpd1* mutant whereas a concomitant decompaction event is observed for 5S rDNA from chromosome 4 and 45S loci in *nrpe1* and *nrpd2* mutants. This might illustrate a common regulation, Pol V-mediated, at 45S and adjacent 5S rDNA.

### RdDM qualitatively controls 5S-210 RNA transcription

The quantitative 5S RNA derepression associated with chromatin decompaction is unambiguously limited to the 5S array from chromosome 4 in PolV-loss of function mutants. However, previous results have unequivocally shown that 5S rDNA is hypomethylated in NRPD1, RDR2 and DRM2 mutants of the RdDM pathway [4],[5],[11],[24],[35]. 5S rDNA loci consist of both active and heterogenous-silent copies of the 5S RNA gene. We previously showed that the release of silencing of 5S RNA genes illustrated by the increase of the proportion of “heterogenous” 5S transcripts (*i.e.* containing some mutations in the genic region) can occur without changes of the 5S RNAs quantity [30],[36].

We therefore decided to analyze the heterogeneity of 5S-210 transcripts produced by 5S arrays from chromosomes 4 and 5 to test whether NRPD1, RDR2, DRM2 and NRPE1 act on each 5S array. As shown Table 1, the proportion of heterogenous 5S RNA from chromosomes 4 and 5 is enhanced in *nrpd1*, *nrpe1*, *nrpd2*, *rdr2* and *drm2* compared to the WT. It demonstrates the impact of Pol IV, RDR2, DRM2 and Pol V on the 5S array from both chromosomes.

**Table 1:** Proportion of heterogenous 5S-210 transcripts (%) from chromosomes 4 and 5.

	from Chr. 4	from Chr. 5
**WT**	5.7	2.9
***nrpd1***	20.8^a^	13.2^c^
***nrpe1***	30.9^a^^, ^ b	11.8^c^
***nrpd2***	19.6^a^	13.7^c^
***rdr2***	20.5^a^	15.4^c^
***drm2***	15,4	13,5^c^

a Significantly different from WT – Fisher's exact test (P<0.05).

b Not significantly different from *nrpd1*, *nrpd2*, *rdr2* values - Fisher's exact test (P>0.2).

c Significantly different from WT - Fisher's exact test (P<0.05).

The 70, 53, 68, 51, 39 and 52 5S-210 cDNA clones from WT, *nrpd1*, *nrpe1*, *nrpd2*, *rdr2*, and *drm2* plants respectively, were analyzed for the presence of nucleotide substitutions (heterogeneity) in the first 120 bp of their sequence. (An alignment is provided Figure S1).

These results refine our previous conclusions indicating that silencing of 5S RNA genes from chromosome 5 is controlled by Pol IV, RDR2, DRM2 and Pol V at the qualitative level. Their mutation has an equivalent effect *i.e.* a derepression of heterogenous 5S RNA genes is observed without increasing the total amount of 5S-210 RNA. Silencing of 5S RNA genes from chromosome 4 is controlled by Pol IV, RDR2, DRM2 and Pol V at the qualitative level, and Pol V exerts an additional role acting at the quantitative level.

The results show that Pol IV, RDR2, DRM2 and Pol V act in the RdDM pathway, to maintain the repression of heterogenous 5S RNA genes from chomosomes 4 and 5. They also show a specific and additional role of Pol V, Pol IV-, RDR2- and DRM2- independent and therefore RdDM-independent, on 5S array from chromosome 4. There is therefore a differential Pol V impact on 5S arrays from chromosomes 4 and 5.

### Pol V–additional activity is not associated with changes in asymmetric DNA methylation

**Figure 4:**
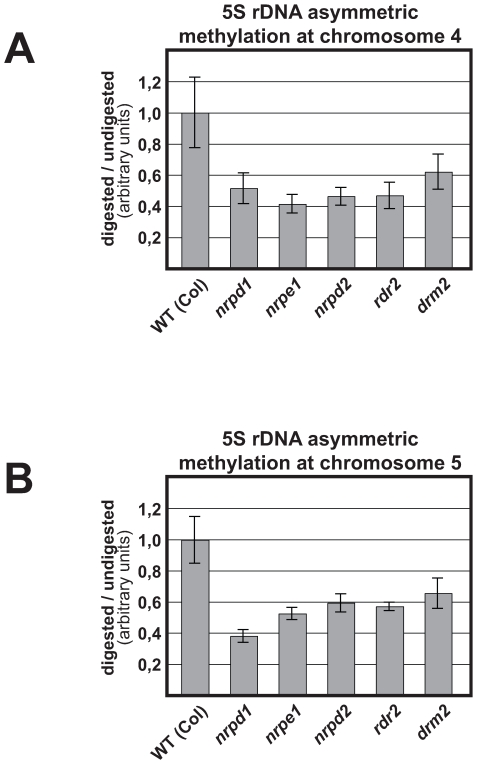
Asymmetric 5S rDNA methylation at chromosomes 4 and 5. DNA methylation assays using a methyl-sensitive restriction enzyme and PCR amplification were specifically performed at chromosomes 4 and 5. PCR amplification on digested (Nla III) and undigested DNA from WT, *nrpd1*, *nrpe1*, *nrpd2*, *rdr2* and *drm2* plants were performed. PCR amplification of *ACTIN 2* was used to control equal templates concentration. Total digestion was controlled with *APETALA1* gene which contains 2 non methylated NlaIII sites. For each experiment, the DNA methylation level was calculated with the ratio: Amount of amplicon in digested DNA/Amount of amplicon in undigested DNA. A graphic representation of 5 to 8 independent experiments is given for (A) chromosome 4 and (B) chromosome 5. The standard deviation of the mean is indicated on each bar.

To confirm that Pol V-additional activity at chromosome 4 is RdDM-independent, we assayed cytosine methylation in asymmetric sequence context (CHH), which largely results from RdDM. Indeed, if the Pol V-additional activity is RdDM-independent, a lower DNA methylation in *nrpe1* and *nrpd2* compared to RdDM mutants is not expected [3],[37],[38]. Digestion with NlaIII for which the same restriction site is present in the intergenic spacer of every 5S rDNA unit of chromosomes 4 and 5 and PCR amplification with primers hybridizing to the chromosome-specific T-stretch were performed. As shown Figure 4, there is a reduction of CHH (CAT for NlaIII) methylation in *nrpd1*, *nrpe1*, *nrpd2*, *rdr2* and *drm2* mutants compared to the WT for both 5S rDNA arrays. Moreover, the same reduction of methylation is observed at chromosome 4 in all the mutants. These results reveal that derepression and decompaction of the 5S array at chromosome 4 in *nrpe1* is not associated with specific changes of 5S rDNA asymmetric methylation. They also show that the similar reduction of methylation observed at both arrays results from the loss of RdDM pathway.

## Discussion

Previous results have shown that 5S rDNA is subject to a variety of overlapping regulation pathways, such as the limiting amount of TFIIIA (TRANSCRIPTION FACTOR IIIA; 5S rDNA specific) [30], the methylation-independent MOM1 pathway [31], the DDM1/MET1- pathway [30],[39], as well as the Pol IV/Pol V RdDM [4],[24],[39]. Although the impact of the Polymerases IV and V on 5S rDNA was previously demonstrated on the basis of DNA hypomethylation, decrease of 5S small RNA accumulation and chromatin decompaction [4],[24],[39], the relative impact of Pol IV and Pol V, their potential selective action on the different 5S arrays and the consequence of their mutation on 5S rDNA silencing of each 5S array were unknown.

In this paper, we have shown that silencing of 5S RNA genes from chromosomes 4 and 5 is controlled at the qualitative level by RdDM including Pol IV, RDR2, DRM2 and Pol V activities. Loss of this silencing pathway leads to the derepression of heterogenous 5S RNA genes, without an increase of total 5S RNA amount and without a detectable chromatin decompaction. These results are consistent with the previously reported reduction or elimination of 5S siRNAs (the 1003 siRNA) as well as with the 5S rDNA hypomethylation observed in *nrpd1*, *rdr2*, *drm2*, *nrpe1*, *ago4* or *dcl3* mutants [4],[5],[11],[24],[40]. Our results demonstrate that each of the two 5S arrays is a target of RdDM. Derepression of heterogenous 5S RNA genes at chromosomes 4 and 5 in mutants of the RdDM pathway is associated with reduction of asymmetric methylation at each array.

In addition to the RdDM process common to 5S arrays from chromosomes 4 and 5, an additional Pol V activity specifically applies to chromosome 4. Higher amounts of 5S-210 transcripts from chromosome 4 are observed in *nrpe1* and *nrpe5a*, two Pol V-specific subunits, correlating with a specific decompaction of this 5S rDNA array in *nrpe1* and *nrpd2*. On the contrary, similar amounts of transcripts were obtained in WT and all the tested RdDM mutants, and chromatin decompaction is absent in *nrpd1*. Therefore, the additional role of Pol V on chromosome 4 is Pol IV- and RdDM- independent. Moreover, the Pol V activity observed specifically at chromosome 4 is not associated with changes of 5S rDNA asymmetric methylation, in agreement with the similar global 5S rDNA methylation observed in *nrpd1* and *nrpe1* (symmetric and asymmetric methylation; [5]. It suggests that Pol V complex might recruit other repressive epigenetic marks than DNA methylation and probably also other than H3K9me2, since 5S rDNA is not decompacted in *met1* mutants [36], despite a decrease of H3K9me2 [42]. H3K27me2, a repressive mark methylation-independent, which labels 5S rDNA [41], is a potential candidate.

The release of silencing of 5S RNA genes that we observed in RdDM mutants without changes of the 5S RNAs quantity might be surprising. However, we previously observed this phenomenon in several mutants [36]. Moreover, in *S. cerevisiae* the number of active rRNA genes can change more than twofold without changing steady-state rRNA transcript levels [43]. On the contrary, higher amounts of 5S-210 transcripts from chromosome 4 are observed in *nrpe1*and *nrpe5a*, suggesting that the large 5S rDNA decompaction enhances the transcription *i.e.* it might facilitate the access to transcription factors and/or the reinitiation process.

The needs for stoichiometric amounts of 5S and 45S rRNA implies that co-regulation events apply to 5S and 45S rDNA. We have shown that NOR loci and 5S array from chromosome 4 are decompacted in *nrpe1* and *nrpd2* mutants illustrating a common regulation process. This Pol V activity, NRPD1 and RdDM-independent, suggests that an alternative pathway *i.e.* without NRPD1 and RdDM partners, exists to target 5S and 45S rDNA loci.

Our results open the question about the interest for the plant to have a 5S array whose regulation is more dependent on Pol V. There is a need of the plant kingdom for rapid, reversible changes in gene expression, to respond to growth demands or environmental changes. Some of the rDNA repeats may be specifically targeted for silencing as a mechanism to modulate or fine-tune total cellular rDNA gene activity.

In conclusion, our results provide new insights on Pol V function in 5S rDNA regulation. On the basis of previous results [4],[5],[11],[24] it was supposed that RdDM (including Pol IV, RDR2 and Pol V activities) was responsible for both silencing and compaction of 5S RNA genes. The current study confirms the participation of both Pol IV and Pol V in the 5S RNA gene silencing of each array, and clearly shows that 5S RNA silencing and compaction are not necessarily linked. It demonstrates that 5S rDNA decompaction is due to the sole Pol V- loss of function, and not to that of both Pol IV and Pol V. Finally, it shows that Pol V has two different activities on the same 5S array, one in the RdDM pathway and the other one, RdDM-independent and chromatin-based which is not associated with methylation changes in early development.

## Materials and Methods

### Plant material

*Arabidopsis thaliana nrpd1a-1 (nrpd1)*, *nrpd1b-1 (nrpe1)*, *nrpd2a-1* (*nrpd2*), *rdr2-1*, *dcl3-1*, *drm2-2* and the corresponding wild type young plantlets were from Columbia ecotype. *nrpd1a-1*, *nrpd1b-1*, *rdr2-1*, *dcl3-1* seeds were obtained from Dr T. Lagrange (University of Perpignan, France). *nrpd2a-1* and *drm2-2* seeds were obtained from the Arabidopsis Biological Resource Center (Stock # SALK 095689 and 150863 respectively). *sil1*, *ago4-1* and WT plants were in *Landsberg erecta* background. Seeds of WT, *sil1* and *ago4-1* plants were obtained from the NASC (stock numbers NW20 for Ler-0, N1894 for *sil1* and N6364 for *ago4-1*). *nrpe5a-1* and WT WS seeds were obtained from Dr T. Lagrange. After synchronization 2 days at 4°C, seeds were grown on a germination medium (MS Salt [Duchefa Biochemie] supplemented with 3% sucrose and 0.8% BactoAgar) in a growth chamber using a 16 h light (120 µE.m^−2^.sec^−1^)/8 h dark regime at 23°C. Plantlets at 4 days post-germination were used.

### Fluorescent *in situ* hybridization (FISH)

Prior to use, tissues were fixed in ethanol/acetic (3∶1) solution. Probes were labeled by PCR using gene specific primers with biotin-16-UTP (Roche) or digoxigenin-11-UTP (Roche). FISH experiments were performed according to Mathieu et al. (2003). Biotin-labeled (5S rDNA) and digoxigenin-labeled (25S rDNA) probes were used. Avidin conjugated with Texas Red (1∶500; Vector Laboratories) followed by goat anti-avidin conjugated with biotin (1∶100; Vector Laboratories) and avidin–Texas Red (1∶500) were used for the detection of the biotin-labeled probe; mouse anti-digoxigenin (1∶125; Roche) followed by rabbit anti-mouse fluorescein isothiocyanate (FITC) (1∶500; Sigma) and Alexa 488-conjugated goat-anti-rabbit (Molecular Probes) were used for the detection of the digoxigenin-labeled probe. Before microscopic analysis, nuclei were stained with DAPI (4′, 6-diamidino-2-phenylindole).

### Microscopy and image processing

For microscopic analysis, an epifluorescence Imager Z1 microscope (Zeiss) with an Axiocam MRm camera (Zeiss) was used. Fluorescence images for each fluorochrome were captured separately through the appropriate excitation filters. The images were pseudocolored, merged and processed with the Adobe Photoshop software (Adobe Systems). 45 to 62 nuclei were analyzed for each genotype.

Compaction of 5S arrays from chromosomes 4 and chromosomes 3+5 were considered separately. Each group of 5S array (4 or 3+5) was considered as decompacted when at least one signal was decondensed. The number of NOR signals was analyzed in 45 to 62 nuclei for each genotype.

### Statistical analysis

Proportion of 5S-210 transcripts from chromosomes 4 and 5 and percentage of heterogenous 5S-210 transcripts were compared with Fisher's exact test for a 2×2 contingency table. The probabilities were calculated from a one-tailed test. Statistical analysis of 5S-210 transcripts amounts were performed using the nonparametric Mann-Whitney U-test with mean values comparison. For statistical analyses of 5S rDNA and NOR compaction, a comparison of proportions Z-test was used. The probabilities were calculated from a one-tailed test. Interval confidence (IC) was calculated for each proportion with a confidence level of 99%.

### RT–PCR analysis

Aliquot of 1 µg of total RNA was treated with DNA-free™ Kit (Ambion) and 100 ng of DNase-treated total RNA was used as input in semi-quantitative RT-PCR reactions using the OneStep RT-PCR Kit (Qiagen). Controls were performed without reverse transcription step to detect contaminating DNA. Amplification of *ACTIN2* RNA was used as an internal control and to normalize RNA amounts. Detection of 5S-210 and *ACTIN2* transcripts was performed in the same reaction tube. Amplification conditions: 50°C for 30 min (reverse transcription step); 95°C for 15 min (reverse transcriptase inactivation step); 30 cycles [95°C for 30 s; 51°C for 30 s; 72°C for 45 s]; 72°C for 10 min. 5S-210 transcripts were amplified using primers RTPCR5S1 (5′-GGATGCGATCATACCAG-3′) and 5SUNIV2 (5′-CGAAAAGGTATCACATGCC-3′). *ACTIN2* transcripts were amplified using primers ACT2-F and ACT2-R according to Vaillant et al. [36].

Amounts of amplicons were estimated using a Versadoc coupled to the QuantityOne software (Biorad).

### Subcloning and sequencing

PCR products were subcloned in the pGem-T easy plasmid using the pGem-T vector system (Promega). Sequencing was performed using the CEQ 2000 Dye terminator cycle sequencer (Beckman). Computer sequence analysis was performed with the Clustawl program (www.infobiogen.fr).

### Genomic DNA extraction and methylation detection assays

Genomic DNA was extracted from seedlings according to the cetyltrimethylammonium bromide (CTAB) method [44]. 200 ng of DNA was digested with NlaIII, which recognizes the sequence CATG and is inhibited by methylation of the cytosine, overnight at 37°C. PCR amplification was subsequently done on 20 ng of digested and undigested DNA using the following primers: CATCCCTC(T)17 specific for chromosome 4, CATCCCTCTTTTATGTTTAACC specific for chromosome 5 and TCGAAAACAATGCTTGAACAAG used for both arrays. The specificity of this amplification was tested on YACs containing respectively the 5S array from chromosome 4 (YAC 9D3) and from chromosome 5 (YAC 6A1) [45].

Primers ACT2-F and ACT2-R Vaillant et al. [36] were used to amplify *ACTIN2*. *ACTIN2* amplification was used to control equal templates concentration.

Total digestion was controlled with *APETALA1* gene (accession AT1G69120.1) which contains 2 non methylated NlaIII sites. Primers: 2F: TTTGGTTGGTTCAGATTTTGTTTCG and 2R: CCAAGAATCAGTGGAGTATTCGAAG were used for PCR amplification.

Amplification conditions: 20 cycles [95°C for 30 s; 60°C for 30 s; 72°C for 30 s]; 72°C for 10 min for 5S rDNA. *ACTIN2*: 28 cycles [95°C for 30 s; 55°C for 30 s; 72°C for 30 s]; 72°C for 10 min. *APETALA1*: 25 cycles [95°C for 30 s; 58°C for 30 s; 72°C for 30 s]; 72°C for 10 min. 5 to 8 experiments were performed.

Amounts of amplicons were estimated using a Versadoc coupled to the QuantityOne software (Biorad). The DNA methylation level was calculated with the ratio: Amount of amplicon in digested DNA/Amount of amplicon in non-digested DNA.

## Supporting Information

Figure S1 Sequence alignment of 5S-210 transcripts. Representative sequence alignment of heterogenous 5S-210 transcripts from chromosomes 4 and 5 with the reference sequence from the same chromosome. Nucleotide positions diverging from the reference are in black box. The chromosome-specific T-stretch is in grey.
(2.91 MB EPS)
